# Expression of MicroRNAs Is Dysregulated by HIV While *Mycobacterium tuberculosis* Drives Alterations of Small Nucleolar RNAs in HIV Positive Adults With Active Tuberculosis

**DOI:** 10.3389/fmicb.2021.808250

**Published:** 2022-02-22

**Authors:** Oskar Olsson, Fregenet Tesfaye, Rolf Søkilde, Jolanta Mazurek, Markos Abebe, Habtamu Yeba, Abraham Aseffa, Sten Skogmar, Taye Tolera Balcha, Carlos Rovira, Per Björkman, Marianne Jansson

**Affiliations:** ^1^Clinical Infection Medicine, Department of Translational Medicine, Lund University, Malmö, Sweden; ^2^Department of Infectious Diseases, Skåne University Hospital, Malmö, Sweden; ^3^Armauer Hansen Research Institute, Addis Ababa, Ethiopia; ^4^Breastca-Genetics, Cancer and Non-coding RNA, Lund University Cancer Centre, Lund University, Lund, Sweden; ^5^Department of Medicine Huddinge, Karolinska Institutet, Stockholm, Sweden; ^6^Adama Public Health Research and Referral Center, Adama, Ethiopia; ^7^Division of Medical Microbiology, Department of Laboratory Medicine, Lund University, Lund, Sweden

**Keywords:** HIV, tuberculosis, small non-coding RNA, microRNA, small nucleolar RNA, anti-tuberculosis treatment, anti-retroviral therapy

## Abstract

HIV infection affects the course of tuberculosis (TB), and HIV and *Mycobacterium tuberculosis* (Mtb) synergize in disease progression through complex immunological interplay. To gain further understanding of these mechanisms, we compared the microRNA (miRNA) and small nucleolar RNA (snoRNA) expression patterns in whole blood of individuals with active TB, with and without HIV coinfection (HIV+/TB+ and HIV-/TB+), and HIV and TB-negative individuals (HIV-/TB-). We found that 218 miRNAs were differentially expressed between HIV+/TB+ and HIV-/TB+, while no statistically significant difference in snoRNA expression was observed between these groups. In contrast, both miRNA (*n* = 179) and snoRNA (*n* = 103) expression patterns were significantly altered in HIV+/TB+ individuals compared to those of the HIV-/TB- controls. Of note, 26 of these snoRNAs were also significantly altered between the HIV-/TB+ and HIV-/TB- groups. Normalization toward the miRNA and snoRNA expression patterns of the HIV-/TB- control group was noted during anti-TB and antiretroviral treatment in HIV+/TB+ participants. In summary, these results show that HIV coinfection influences miRNA expression in active TB. In contrast, snoRNA expression patterns differ between individuals with and without active TB, independently of HIV coinfection status. Moreover, in coinfected individuals, therapy-induced control of HIV replication and clearance of Mtb appears to normalize the expression of some small non-coding RNA (sncRNA). These findings suggest that dysregulation of miRNA is a mechanism by which HIV may modify immunity against TB, while active TB alters snoRNA expression. Improved understanding of how regulation of sncRNA expression influences the disease course in coinfected individuals may have implications for diagnostics, risk stratification, and host-directed therapy. Here, we propose a novel mechanism by which HIV alters the immune response to TB.

## Introduction

HIV infection is the strongest known risk factor for active tuberculosis (TB) (Ai et al., 2016). Furthermore, TB is the leading cause of death among people with HIV (PWH), causing one-third of HIV-related deaths globally (UNAIDS, 2019).^1^ The pathogenesis of active TB is largely determined by interactions with different components of the immune system, and several such mechanisms have been shown to be involved in the dysregulated immune responses observed in HIV/TB coinfection (Pawlowski et al., 2012). Previous studies have described HIV-related alterations in apoptosis (Patel et al., 2007), antigen presentation (Singh et al., 2016), immune cell functionality (Kalsdorf et al., 2009), and cytokine release (Zhang et al., 1994) in the response to TB.

Small non-coding RNAs (sncRNAs), like microRNAs (miRNAs) and small nucleolar RNAs (snoRNAs), have been implicated in regulating immune responses (Okuyama et al., 2014). miRNAs can affect both innate and adaptive immune responses through post-transcriptional regulation of a vast number of protein-coding genes by suppressing mRNAs, including those encoding proinflammatory cytokines (O’Connell et al., 2010). In addition, several bacteria and viruses, including Mtb and HIV, modify inflammatory responses via miRNA dysregulation (Drury et al., 2017). Studies have demonstrated mechanisms by which HIV (Kapoor et al., 2015) and Mtb (Sahu et al., 2017) manipulate specific miRNAs to evade the immune response. In contrast, snoRNAs regulates protein synthesis through post-transcriptional chemical modifications of rRNA (Cao et al., 2018). snoRNA expression is essential for the infectivity of several viruses (Stamm and Lodmell, 2019). Moreover, snoRNA expression differs among individuals with active and latent TB infection (de Araujo et al., 2019). However, the impact of HIV coinfection on the expression of these sncRNAs in individuals with active TB has not been explored.

Thus, to investigate the role of sncRNAs in the pathogenesis of active TB in PWH, we analyzed expression patterns of miRNAs and snoRNAs in whole blood samples of individuals with active pulmonary TB, with or without HIV coinfection (HIV+/TB+ and HIV-/TB+, respectively), and in HIV-negative individuals without TB infection (HIV-/TB-). Furthermore, to explore the effects of antiretroviral (ART) and antituberculosis (ATT) treatments on sncRNA expression, we studied miRNA and snoRNA expression patterns longitudinally before and after therapy initiation.

## Materials and Methods

### Selection of Study Participants and Clinical Sampling

Participants for this study were recruited and followed at public health centers providing TB and HIV care in and around the city of Adama, Oromia Region, Ethiopia, between 2011 and 2015. Three groups of participants were included in this study. First, HIV-positive adults (age ≥18 years) with pulmonary TB (HIV+/TB+) were selected from a cohort of PWH who had been bacteriologically characterized for active TB (Balcha et al., 2014). These participants were required to have bacteriologically confirmed pulmonary TB (by liquid culture, direct microscopy, and/or Xpert MTB/RIF assay), to not have received ATT within the preceding six months, to not currently be receiving ATT for more than two weeks, and to not have received ART. ATT was initiated when a positive bacteriological result was known and subsequently also non-nucleoside transcriptase-inhibitor-based ART, both following the Ethiopian guidelines (Federal HIV/AIDS Prevention and Control Office Federal Ministry of Health, 2008).^2^ Second, HIV-negative adults with pulmonary tuberculosis (HIV-/TB+), diagnosed by sputum smear microscopy or clinical criteria, were identified at TB clinics at the same health facilities. The exclusion criteria applied were confirmed or suspected extrapulmonary TB, ATT within the previous 6 months and/or treatment for the current episode of TB for > 2 weeks, and presence of a chronic disease. Third, healthy HIV-negative subjects without active or latent TB infection (HIV-/TB-) were recruited during voluntary counseling at HIV testing clinics (at the same facilities). To exclude TB infection, a negative result for QuantiFERON Gold-in-Tube test (<0.35 IU/mL), and the absence of symptoms suggestive of active TB were required. Blood samples were collected from all participants at the time of enrollment, and additional sampling was scheduled at six months after the initiation of ATT for HIV-/TB+ subjects and initiation of ART for HIV+/TB+ subjects.

Venous blood samples were collected in syringes and directly transferred to PAXgene^®^ Blood RNA Tubes (BD Biosciences, San Jose, CA, United States). PAXgene tubes were subsequently frozen at −80°C and transported with an intact cold chain to Lund University, Sweden, for further analysis. In addition, blood samples were collected for determining total blood count (Sysmex KX-21; Sysmex Corporation, Kobe, Japan) and CD4^+^ cell count by flow cytometry (FACS Calibur; Becton Dickinson, Franklin Lakes, NJ, United States) for all participants and for viral load determination in HIV+/TB+ patients. Plasma viral load was determined using the Abbott m 2000rt RealTime system (lower detection limit 40 RNA copies/mL; Abbott, Chicago, IL, United States).

### sncRNA Expression Analysis Using Microarray

RNA was extracted from whole blood using the PAXgene Blood miRNA Kit (Qiagen, Hilden, Germany) according to the manufacturer’s instructions. RNA concentrations and purity were assessed using a NanoDrop 2000 spectrophotometer (NanoDrop Technologies, Wilmington, DE, United States). Samples with low RNA concentrations (< 30 ng RNA/μl) were concentrated using a SpeedVac vacuum concentrator. Before microarray analysis, RNA integrity was assessed using Agilent 2100 Bioanalyzer (Agilent Technologies). The RNA integrity number was calculated based on 28S and 18S rRNA peaks and the absence of degradation products. Samples with RNA integrity numbers > 7 were included in the further analysis. Samples (370 ng of RNA) were analyzed for sncRNA expression on the Affymetrix Platform, GeneChip^®^ miRNA 4.0 Array (Affymetrix, Thermo Fisher Scientific, Waltham, MA, United States) using 4603 probes for human miRNA (including mature miRNA and stem-loop precursors) and 1996 probes for human snoRNA (including snoRNA, CDBox, HAcaBox, and small Cajal-body RNA (scaRNA) at the Swegene Centre for Integrative Biology (SCIBLU), Lund University, Sweden.

### Determination of miRNA and snoRNA Expression Using Quantitative PCR

For cDNA synthesis, 4 ng/μL RNA template was poly-A-tailed with *E. Coli* poly-A polymerase and reverse-transcribed using MuLV reverse transcriptase (both enzymes were obtained from New England Biolabs Inc., Ipswich, MA, United States). miRNA-39 from *C. elegans* was spiked in as an internal control. PCR was performed with cDNA at 1/20 dilution, using forward and reverse primers (for primer sequences see Supplementary Table 1), designed using the miRprimer software (Busk, 2014). SYBR Green dye (Applied Biosystems™, Thermo Fisher Scientific, Waltham, MA, United States) was used for quantitation. RT-qPCR was performed on the StepOnePlus™ real-time PCR system (Applied Biosystems™). The reaction mixture was heated to 95°C for 15 min and then the temperature was altered between 95°C (15 s) and 60°C (30 s) for 45 cycles. All reactions for each sncRNA were carried out in a single 96-well plate to minimize technical variation. Melting curves from 60 to 99°C were determined to ensure specificity. Each plate included two negative controls, one without template and one without reverse transcriptase. Each plate included a positive control sample of human reference RNA.

### Data Analysis and Statistics

All data analyses were performed using RStudio version 1.3.959 (Boston, MA, United States). The microarray results were normalized using the robust multi-array average method (Irizarry et al., 2003), and the data were log_2_ transformed. The microarray chip contained probes for 6599 human sncRNA targets. Probes that generated detection above background *P* > 0.01 in > 20% of samples were filtered out. In the cases where several probes represented the same RNA, results from the probe with the highest expression values were chosen. miRNAs that had been removed from the miRBase database were removed from further analysis. This filtering rendered inclusion of 674 sncRNAs for further analysis. Differential expression of miRNAs and snoRNAs was assessed using the R package Limma (Ritchie et al., 2015). sncRNAs with the Benjamini-Hochberg false discovery rate (FDR) was less than 0.05, were considered significantly differentially expressed. The microarray data were analyzed using principal component analysis (PCA), in which the data were median centered, and all samples were plotted on a graph with principal components 1 and 2 as the axes. Differences between the groups in principal components 1 and 2 were assessed using Hotelling’s T-squared test. In addition, hierarchical clustering was performed, for the 20 most differentially expressed (smallest adjusted *P*-value) from each contrast, using the Pearson correlation as the distance measure, and average linkage to build clusters.

For the RT-qPCR, we used exogenous spike-in cel-miR-39 as an internal reference. Fold change values were calculated using the double delta method, and statistical significance was assessed using the Mann-Whitney U test for the comparisons between groups before treatment and Wilcoxon signed-rank test for the comparisons of paired samples before and after treatment. Holm-Bonferroni correction was applied to adjust for multiple testing (correcting for the number of RNAs that were tested in each group comparison). For easy interpretation, the *P*-values were adjusted rather than the significance threshold, and all *P*-values reported in the qPCR results section were adjusted using the Holm-Bonferroni method. To assess how different RNAs correlated with CD4^+^ cell count, Spearman’s rank correlation analysis was performed with Holm-Bonferroni correction for number of tests.

## Results

### Participant Characteristics

A total of 40 participants were included [HIV+/TB+ (*n* = 13), HIV-/TB+ (*n* = 13), and HIV-/TB- (*n* = 14)], and the majority were women, irrespective of infection status (Table 1). At inclusion, all HIV+/TB+ subjects were ART naïve. The median baseline viral load was 5.1 log_10_ RNA copies/mL and the median CD4^+^ cell count was 270 cells/μl in coinfected individuals. In 12 HIV+/TB+ subjects, ART was started after ATT (median 39 days, range 26–141 days), and in one of these subjects, ATT was started after ART (25 days).

**TABLE 1:** Study participant characteristics^a^.

	HIV+/TB+*^b^*	HIV-/TB+*^c^*	HIV-/TB-
Participants	13	13	14
Female	9 (69)	10 (77)	12 (86)
Age (years)	32 (28–45)	28 (23–35)	23 (20–27)
CD4^+^ cell count (cells/μL)	270 (157–328)	639 (494–842)	720 (671–828)
Viral load (log_10_ copies/mL)	5.1 (4.6–5.5)	NA	NA
Hemoglobin (g/dL)	11.8 (10.7–12.4)	12.1 (11.6–12.9)	14.3 (12.6–15.1)
Lymphocyte count (cells/μL)	1,600 (1,100–2,500)	1,400 (1,300–1,650)	1,900 (1,600–2,600)
Neutrophil count (cells/μL)	3,100 (1,900–3,700)	7,000 (3,400–9,825)	3,400 (2,650–4,025)
Platelet count (cells/mL)	242 (195–267)	396 (356–470)	234 (214–265)

*^a^Categorical variables are shown as absolute numbers, with the percentage of the total number given in brackets; continuous variables are given as a median value, with interquartile range in brackets.*

*^b^Of the HIV+/TB+ participants (three were sputum microscopy positive, nine were GeneXpert positive, and one was only culture positive).*

*^c^Among the HIV–/TB+ participants, nine were sputum microscopy positive and four were diagnosed based on clinical criteria. NA, not applicable.*

### Differential Expression of Both miRNAs and snoRNAs in HIV-Infected Individuals With Active Tuberculosis Revealed by Microarray Analysis

**FIGURE 1:**
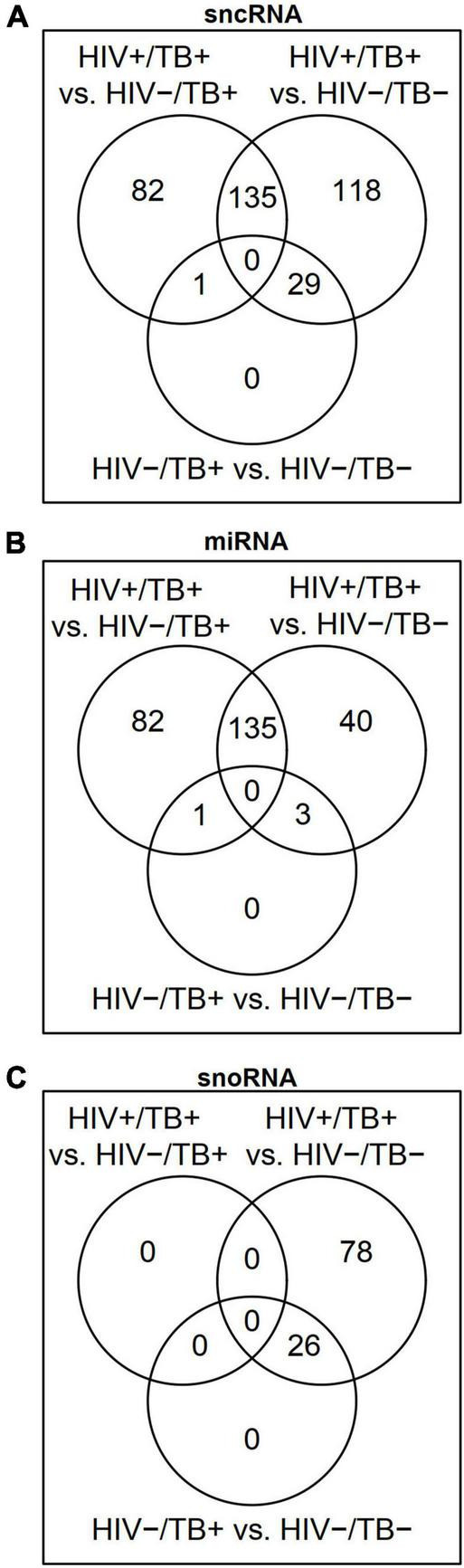
Venn diagrams showing differentially expressed (FDR < 0.05) **(A)** small non-coding RNAs (sncRNAs), **(B)** microRNAs (miRNAs), and **(C)** small nucleolar RNAs (snoRNAs) in the HIV+/TB+, HIV-/TB+ and HIV-/TB- groups.

**FIGURE 2:**
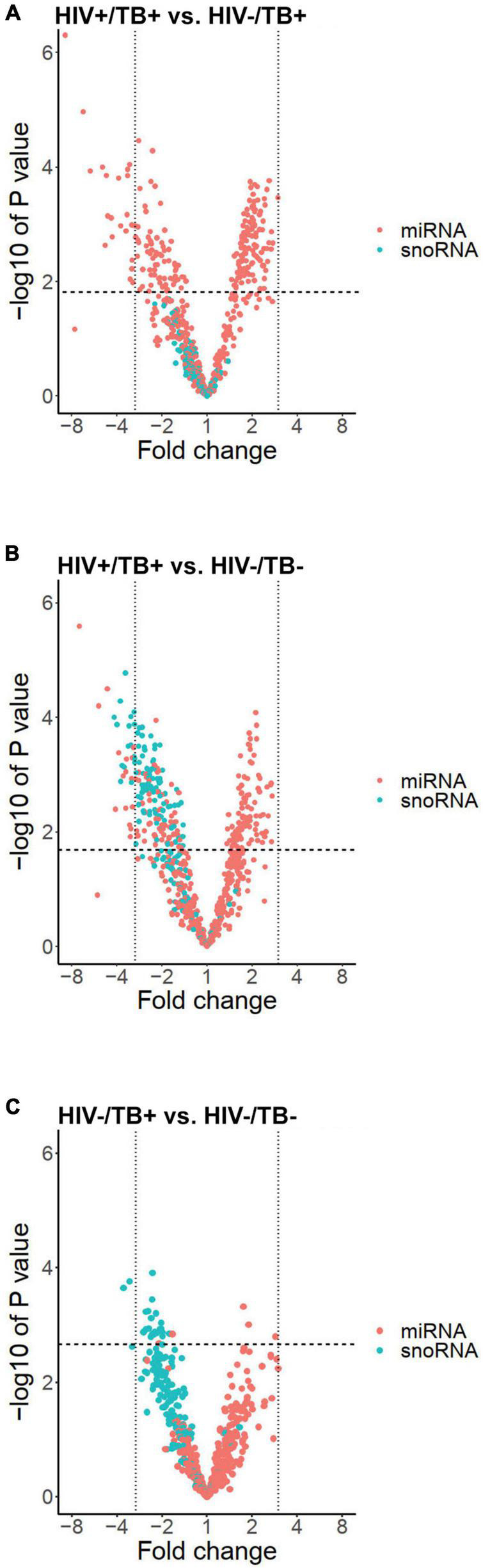
Volcano plots illustrating expression profiles of miRNAs (red dots) and snoRNAs (blue dots) according to fold change on a logarithmic scale (x-axis) and -log of *P*-value (y-axis) in the groups **(A)** HIV+/TB+ vs. HIV-/TB+, **(B)** HIV+/TB+ vs. HIV-/ TB-, and **(C)** HIV-/TB- and HIV-/TB+. Vertical black lines indicate a fold change of three. Horizontal line indicates largest *P*-value generating FDR < 0.05.

To analyze the differential sncRNA expression between HIV+/TB+, HIV-/TB+, and HIV-/TB-, we performed microarray analysis on whole blood samples obtained from seven HIV+/TB+, eight HIV-/TB+, and eight HIV-/TB- subjects. A total of 218 sncRNAs were differentially expressed [false discovery rate (FDR) < 0.05] between HIV+/TB+ and HIV-/TB+ groups (Figure 1A and Supplementary Table 2). All of these sncRNAs were either mature miRNA (*n* = 206) or miRNA precursors (*n* = 12, Figure 1B). In contrast, none of the snoRNAs were differentially expressed between these groups (Figures 1C, 2A). Although the majority of the miRNAs differentially expressed between HIV+/TB+ and HIV-/TB+ were upregulated in HIV+/TB+ subjects, the downregulated miRNAs generally showed a larger magnitude in fold-change (Figure 2A). For example, 22 miRNAs with downregulated expression exhibited a greater than three-fold change, while none of the miRNAs with upregulated expression displayed such a change. Moreover, a larger proportion [79/96 (82%)] of the miRNAs with significantly downregulated expression had high annotation confidence (according to miRbase^3^, the primary miRNA sequence repository), indicating lower rates of false-positive miRNA (Alles et al., 2019), compared to miRNAs with upregulated expression [16/122 (13%)]. Taken together, these results indicate that miRNA expression is significantly altered by HIV coinfection in individuals with active TB.

Besides sncRNAs that were differentially expressed between HIV+/TB+ and HIV-/TB+, 282 sncRNAs, including 179 miRNAs, were differentially expressed comparing the HIV+/TB+ and the control HIV-/TB- subjects (Figures 1A,B and Supplementary Table 2). An overlap (*n* = 135) in miRNAs differentially expressed between HIV+/TB+ group and HIV-/TB+ or HIV-/TB- groups, was noted, as illustrated by the Venn diagram (Figure 1B). Differential expression was in the same direction for HIV+/TB+ compared to both HIV-/TB+ and HIV-/TB- for all these miRNAs. In contrast to the HIV+/TB+ vs. HIV-/TB+ comparison, snoRNA (*n* = 103) expression differed between HIV+/TB+ and HIV-/TB- (Figures 1C, 2B). All differentially expressed snoRNAs were downregulated in HIV+/TB+ subjects (Figure 2B). In the HIV-/TB+ vs. HIV-/TB- comparisons, the 30 differentially expressed sncRNAs were dominated by the 27 snoRNAs (Figures 1A–C), which were also significantly downregulated (Figure 2C). This pattern suggests that active TB predominantly downregulates snoRNA expression, and that this effect is more pronounced in HIV-positive individuals.

### Clustering of Individuals Based on sncRNA Expression

**FIGURE 3:**
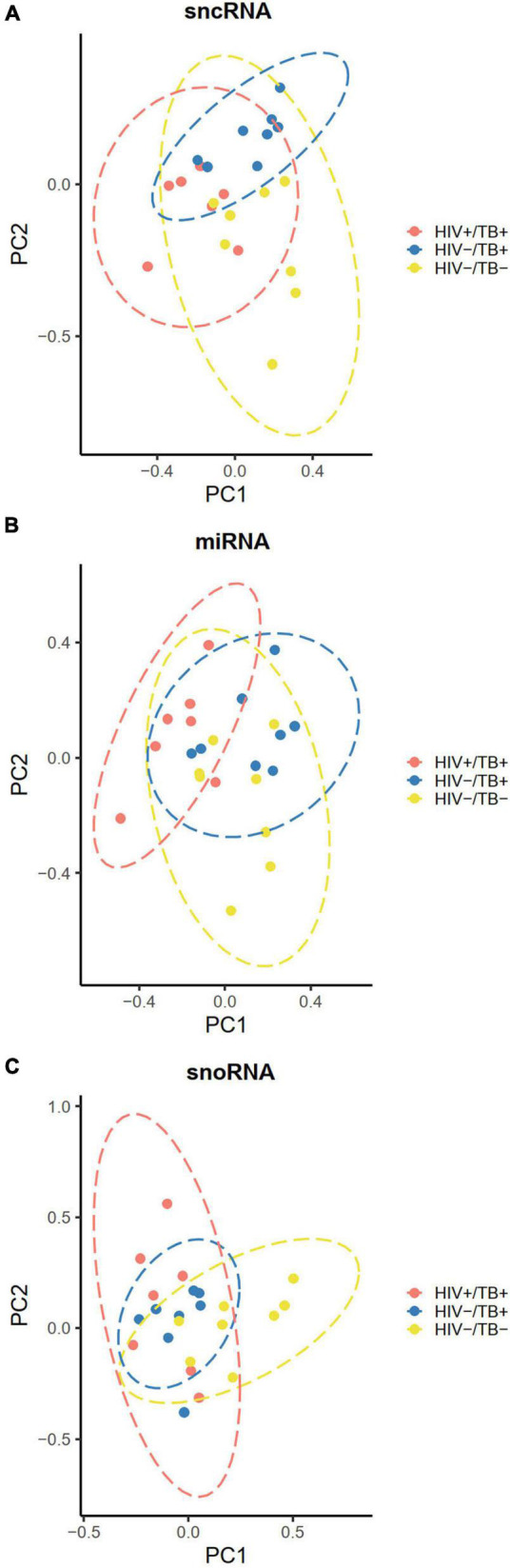
Principal component plots illustrating clustering of the participants according to their infection status HIV+/TB+ (red), HIV-/TB+ (blue) and HIV-/TB- (yellow) based on **(A)** all sncRNAs **(B)** miRNAs **(C)** snoRNAs. Data ellipses calculated based on the t-statistic with 95% confidence intervals. Microarray data were median centered before analysis.

To explore potential clustering of participants based on sncRNA expression profiles, we performed PCA and assessed group differences using Hotelling’s T-squared test. When both miRNA and snoRNA expressions were used for the PCA, significant separation of all three groups based on the sncRNA expression was observed (adjusted *P* < 0.01) (Figure 3A). The separation pattern was less distinct when only miRNA expression was considered for the PCA. The HIV+/TB+ group was significantly different from the other groups (adjusted *P* < 0.01), whereas the separation of the HIV-/TB- and HIV-/TB+ groups was not statistically significant (adjusted *P* = 0.07, Figure 3B). When PCA was carried out for snoRNAs alone, the pattern was markedly different, with no separation of the HIV+/TB+ and HIV-/TB+ groups (adjusted *P* = 0.7), while both these groups were significantly different from the HIV-/TB- group (adjusted *P* < 0.01) (Figure 3C).

**FIGURE 4:**
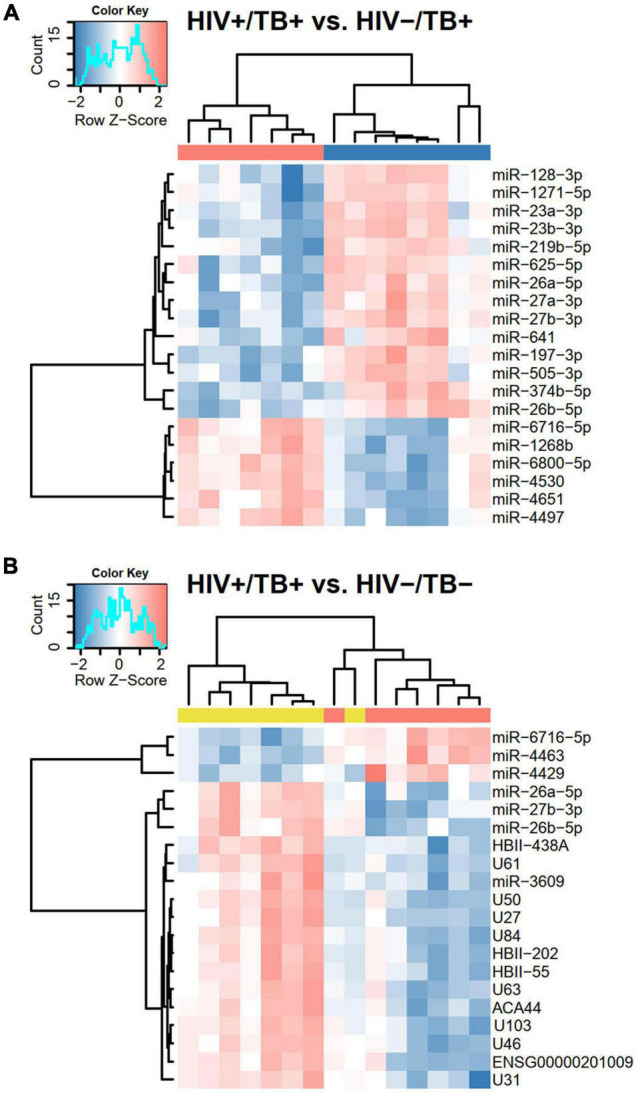
Heatmaps based on hierarchical clustering analyses including **(A)** HIV+/TB+ and HIV-/TB+ and **(B)** HIV-/TB- and HIV+/TB+ subjects and the 20 sncRNAs that generated the smallest *P*-values in Limma analysis from the respective comparisons. Microarray data were median centered before the analysis. Pearson’s correlation was used as a distance matrix. The red and blue colors indicate upregulated and downregulated sncRNAs, respectively. Color codes at the top represent infection status, HIV+/TB+ (red), HIV-/TB+ (blue), and HIV-/TB- (yellow). Probe HBII-438A also assigned to HBII-438B, probe U84 to ENSG00000263442/ENSG00000264591/ENSG00000265325/ENSG00000265607/ENSG00000266646/ENSG00000266755, probe ACA44 to ENSG00000252840, and probe U103 to U103B.

Next, we performed hierarchical clustering of the 20 sncRNAs that generated the lowest *P*-values in pairwise comparisons. With one exception, distinct clustering according to HIV and TB infection status was observed (Figures 4A,B and Supplementary Figure 1). All 20 sncRNAs that showed most significant differential expression between HIV+/TB+ and HIV-/TB+ were miRNAs (Figure 4A). In contrast, in the comparison between HIV+/TB+ and HIV-/TB- (Figure 4B) and the comparison between HIV-/TB+ and HIV-/TB- groups (Supplementary Figure 1), 13 and 18, respectively, of the 20 most significantly differentially expressed sncRNAs were snoRNAs, and all of these were expressed at lower levels in the subjects with active TB. These analyses support the finding that HIV predominantly affects miRNA expression, whereas TB alters the expression of snoRNA.

### Validation of Differential Expression of Small Non-coding RNAs

**FIGURE 5:**
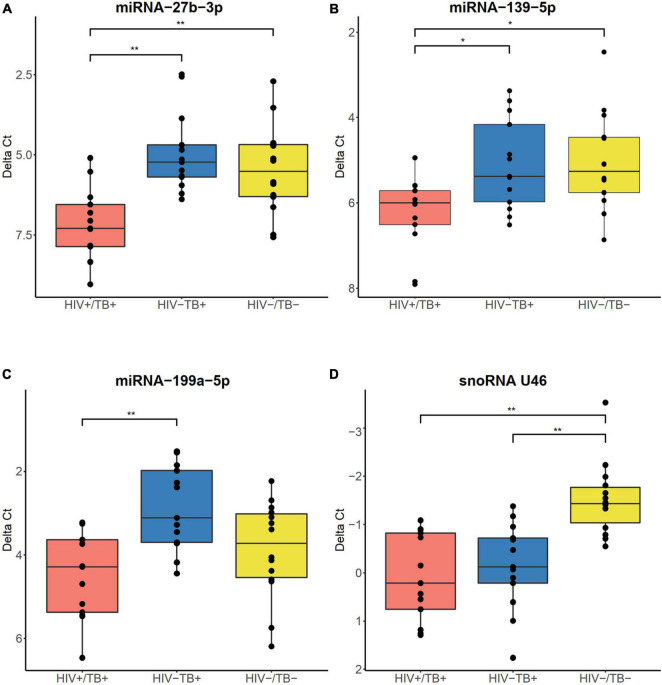
Boxplots illustrating expression of **(A)** miR-27b-3p, **(B)** miR-139-5p, **(C)** miR-199a-5p, and **(D)** snoRNA U46, validated by RT-qPCR. Boxes show median and interquartile range, and whiskers extend to the largest values within 1.5 interquartile range from the quartile. Infection status is represented by colors, HIV+/TB+ (red), HIV-/TB+ (blue), and HIV-/TB- (yellow). Symbols on top indicate: * adjusted *P* < 0.05 and ** adjusted *P* < 0.01. *P*-values from the Mann–Whitney *U*-test with Holm-Bonferroni correction.

To validate the sncRNA expression results obtained from the microarray analysis, we performed reverse transcription quantitative polymerase chain-reaction (RT-qPCR) and expanded the number of study participants, which resulted in the inclusion of a total of 40 individuals (13 HIV+/TB+, 13 HIV-/TB+, and 14 HIV-/TB-). In this validation step, we focused on the expression of three miRNAs (miR-27b-3p, miR-139-5p, and miR-199a-5p), selected based on the microarray results (with FDR < 0.05 and ≥ 3-fold change in expression level), ranging from 3.1 to 8.2-fold down-regulation (Supplementary Table 2) comparing the HIV+/TB+ and HIV-/TB+ groups. The marked decrease of miR-27b-3p and miR-139-5p expression in the HIV+/TB+ group compared to in the other two groups (FC < 0.15 and FC < 0.4, respectively), was validated by qPCR (FC ≤ 0.3, adjusted *P* < 0.01, and FC < 0.5, adjusted *P* < 0.05, respectively, Figures 5A,B). The downregulation of miR-199a-5p expression in HIV+/TB+ compared to HIV-/TB+ (FC = 0.3) was also confirmed by qPCR (FC = 0.3, adjusted *P* < 0.01, Figure 5C). Next, we validated the expression of snoRNA U46, as a representative snoRNA downregulated in the HIV+/TB+ and HIV-/TB+ groups compared to HIV-/TB- subjects from the microarray analysis (FC = 0.3 and 0.4, respectively). This finding was further confirmed using qPCR (FC = 0.3 and 0.4, respectively, adjusted *P* < 0.01, Figure 5D). In addition, we performed correlation analysis between results obtained from the microarray and qPCR for the analyzed sncRNAs, and our results showed that the Spearman’s rank rho was 0.90 for miRNA-27b-3p, 0.94 for miRNA-139-5p, 0.82 for miR-199a-5p, and 0.94 for snoRNA U46 (adjusted *P* < 0.05 for all; Supplementary Figure 2). In summary, microarray findings for the differentially expressed miRNAs and snoRNAs could be validated by qPCR.

### Impact of Antituberculosis and Antiretroviral Treatment on sncRNA Expression

Next, we analyzed the expression of the four sncRNAs (miR-27b-3p, miR-139-5p, miR-199a-5p, and the snoRNA U46) in whole blood samples obtained before and after the initiation of ATT and ART in a subset of the HIV+/TB+ individuals (*n* = 9) for whom the follow-up samples were available. These samples were obtained at a median of 200 days (range 126–331 days) after the initiation of the ATT and at a median of 175 days (range 92–202 days) after starting ART. At follow-up, CD4^+^ cell count was significantly higher than at baseline (*P* = 0.03), and all subjects with available viral load determinations (*n* = 8/9) had undetectable levels.

**FIGURE 6:**
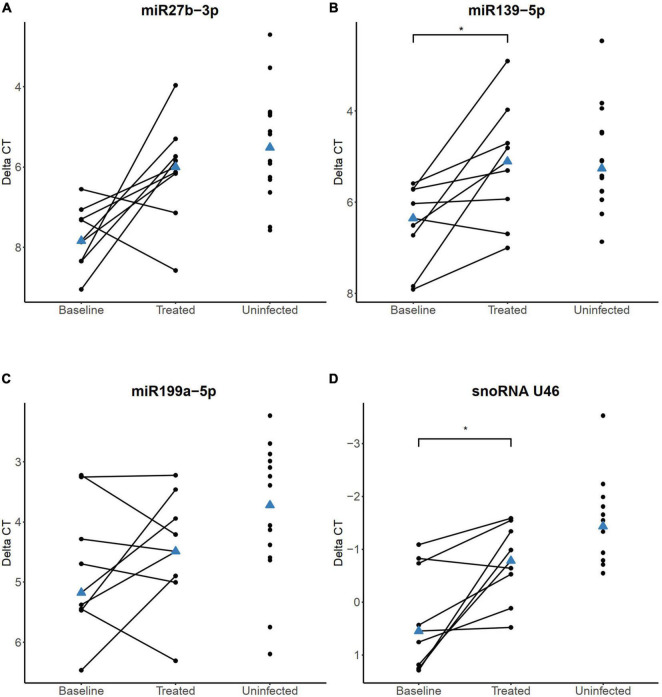
sncRNA expression levels quantified by RT-qPCR in HIV+/TB+ study participants before and after the initiation of anti-tuberculosis treatment and antiretroviral treatment **(A)** miR-27, **(B)** miR-139-5p, **(C)** miR-199a-5p, and **(D)** snoRNA U46. Paired samples of participants longitudinally followed before and during treatment are connected with a line. HIV-/TB- control group is included in all plots for comparison. The blue triangle indicates the median value. *Adjusted *P* < 0.05. *P*-values were calculated from paired samples Wilcoxon test with Holm-Bonferroni correction.

In the follow-up samples, increased expression levels of miR-27b-3p were observed in seven out of nine HIV+/TB+ individuals (FC = 3.1, Figure 6A), however, this change was not statistically significant (adjusted *P* = 0.08). On the contrary, a statistically significant increase in the expression of miR-139-5p was observed during the treatment (FC = 2.5, adjusted *P* = 0.047, Figure 6B). In samples obtained after treatment initiation, the expression of neither miR-27b-3p nor miR-139-5p was significantly different in the HIV+/TB+ group compared to the HIV-/TB- control group. The change in miR-199a-5p expression during treatment varied within the HIV+/TB+ group and no clear trend was observed (Figure 6C). As for the impact of the treatment on snoRNA U46 expression in the HIV+/TB+ group, we observed a significantly increased expression (FC = 2.1, adjusted *P* = 0.047, Figure 6D), which also led to normalization of the expression toward that of the control group.

Follow-up samples from eight HIV-/TB+ participants were obtained after a median of 183 days (range 153–184 days) after starting the ATT. However, we did not find statistically significant changes in any of the four sncRNAs (Supplementary Figure 3), however, the significant alteration of snoRNA U46 was no longer evident in this group when compared to the HIV-/TB- group. In summary, miRNA and snoRNA alterations appeared to normalize during ATT and ART in the HIV+/TB+ group, while clear changes in expression of the analyzed sncRNAs were not seen in the HIV-/TB+ group, in line with less pronounced differential expression before treatment compared to the control group.

### Correlation Analyses Between sncRNA and CD4^+^ Cell Counts at Baseline and During Treatment

**FIGURE 7:**
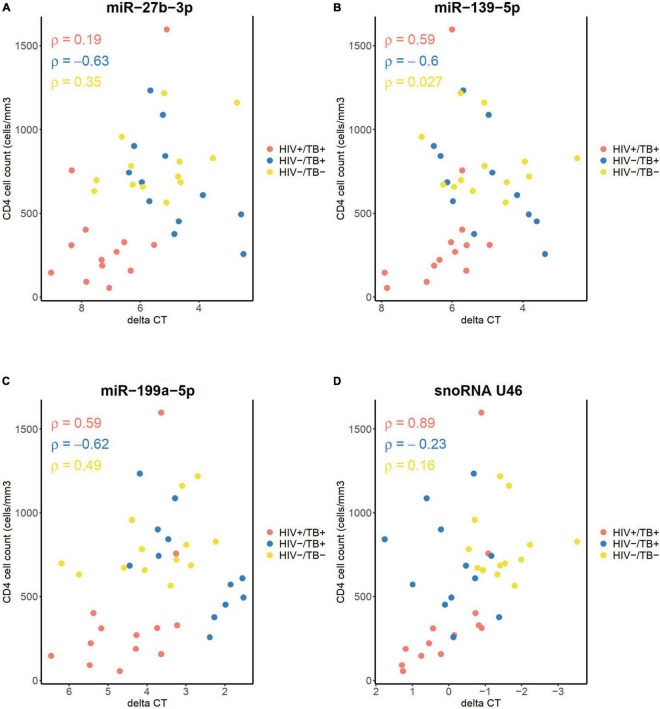
Correlation between CD4^+^ cell count before treatment, and the levels of **(A)** miR-27b-3p, **(B)** miR-139-5p, **(C)** miR-199a-5p, and **(D)** snoRNA U46, measured by qPCR. Infection status is represented by color, HIV+/TB+ (red), HIV-/TB+ (blue) and HIV-/TB- (yellow). Spearman’s rho is indicated for each group in the matching color.

Next, we explored potential associations between expression levels of the sncRNAs and CD4^+^ cell count to assess whether the differences in the sncRNA level in the whole blood between the groups could be due to changes in the whole blood composition, such as depletion of CD4^+^ cells in the coinfected subjects. The three miRNAs measured with qPCR did not show a significant correlation between their expression levels and CD4^+^ cell count in any of the groups, calculated by the Spearman correlation analyses and Holm-Bonferroni adjustments, neither at the baseline (Figures 7A–C) nor at the follow-up time points (data not shown). However, a strong correlation was observed between snoRNA U46 expression and CD4^+^ cell count at the baseline in the HIV+/TB+ subjects (Spearman’s rho 0.89, adjusted *P* < 0.01. Figure 7D). This correlation was not present in samples obtained after treatment initiation (data not shown). No significant correlations were observed between CD4^+^ cell count and any of the miRNAs or snoRNA U46 in the HIV-/TB+ and HIV-/TB- groups. Taken together, these results indicate that peripheral blood CD4^+^ cell count is not the main reason for the differences in expression of the analyzed sncRNA. Moreover, the correlation between snoRNA U46 expression and CD4^+^ cell count was dependent on the infection status and also reversed by ART and ATT in the HIV+/TB+ group, suggesting that other HIV-related mechanisms than CD4^+^ cell count, affects snoRNA U46 expression.

## Discussion

In this study, we demonstrate that HIV coinfection significantly alters miRNA expression during active TB. Our findings also reveal the downregulation of several snoRNAs in subjects with TB, regardless of their HIV infection status. Furthermore, normalization of both miRNA and snoRNA expression levels, toward the levels in the uninfected subjects were observed during ART and ATT in HIV-positive individuals with active TB.

To the best of our knowledge, a comparison of whole blood miRNA and snoRNA expression profiles in individuals with active TB and HIV coinfection has not been reported, and only a handful of studies on miRNA expression in active TB have included the PWH. A recent study, based on a multinational cohort exploring miRNA, metabolites and cytokines as biomarkers for TB in PWH, identified 11 miRNA as differentially expressed between individuals with and without TB (Krishnan et al., 2021). This study, however, was not designed to compare miRNA expression differences between HIV+/TB+ and HIV-/TB+. Two studies (one from Cameroon and one with participants from Italy, Uganda, and Tanzania) included subsets of PWH, but no direct comparisons between patients with and without HIV coinfection were included (Miotto et al., 2013; Ndzi et al., 2019). In addition, Honeyborne et al. (2015) have shown similar changes in total plasma sncRNA levels as an indicator of successful ATT regardless of HIV status, however, comparisons of individual sncRNA expressions between HIV+/TB+ and HIV-/TB+ subjects before treatment were not reported.

Our results clearly show that HIV alters the expression of a range of miRNAs in subjects with active TB. Thus, it is tempting to speculate that these alterations may directly be involved in the insufficient TB control in PWH, since miRNAs reportedly play an important role in multiple immunological responses (Drury et al., 2017). Macrophages play a key role in the interactions between HIV and Mtb (Auld and Staitieh, 2020), as both pathogens infect these cells (Gartner, 2014; Esmail et al., 2018). Moreover, HIV-infected macrophages show increased Mtb growth (Pathak et al., 2010), impaired phagocytosis (Mazzolini et al., 2010), and reduced phagosomal proteolysis function (Jambo et al., 2014). Hence, it is possible that the alterations in miRNA expression in HIV-positive individuals with active TB are linked to impaired macrophage function. Indeed, several of the dysregulated miRNAs identified in our study have known functional roles in macrophages. For example, decreased levels of miR-26a-5p, one of the 20 most downregulated miRNAs in HIV+/TB+ patients in our study, has been linked to decreased nitric oxide synthase activity and prevention of Mtb trafficking to lysosomes (Sahu et al., 2017). miR-23a-3p, found to be downregulated in HIV+/TB+ individuals, was recently shown to be downregulated in cells stimulated with mycobacteria *in vitro* and in peripheral blood mononuclear cells obtained from subjects with TB, leading to impaired generation of reactive oxygen species and phagocytosis (Chen et al., 2020). Furthermore, downregulation of miR-27b-3p (as is reported in these HIV+/TB+ subjects), has been associated with increased survival of Mtb in macrophages and impaired macrophage apoptosis (Liang et al., 2018). In addition, miR-199a-5p, another miRNA found to be downregulated in HIV+/TB+, was demonstrated to have a positive relationship with TLR-4 signaling and IL-6 expression in macrophages in a cystic fibrosis model (Zhang et al., 2015). These findings, taken together with our results, suggest that HIV coinfection may impair the macrophage response to TB.

Chronic immune activation is a hallmark of HIV infection and has been postulated to drive pathogenesis via increased cellular turnover and exhaustion, in addition to dysregulated homing of CD4^+^ and CD8^+^ cells to extra-lymphoid tissues (Moir et al., 2011). Whereas pro-inflammatory responses are required for control of mycobacterial replication, tissue damage is mainly driven by exaggerated immune reactions (Esmail et al., 2018). While the immune-related TB pathology is less prominent at sites of infection in PWH with advanced immunosuppression (manifested by deficient granuloma formation and lower frequency of pulmonary cavitation) (Esmail et al., 2018), systemic inflammation is more prominent (Skogmar et al., 2015), and some studies point toward deficient immunoregulation as a reason for deficient TB control in PWH (Tomlinson et al., 2014; Srabanti Rakshit et al., 2020). The alterations in the expression of certain miRNAs observed in our study have been linked to proinflammatory responses and may contribute to inflammation and chronic immune activation. miR-26b-5p targets TAK1, a component of the proinflammatory NF-κB pathway, suggesting that the downregulation of this miRNA may exert proinflammatory effects (Li et al., 2018). Furthermore, downregulated expression of miR-27b-3p has been linked to the increased activation of CD4^+^ cells (Chiang et al., 2012). Another of the most downregulated miRNAs in HIV+/TB+ individuals, miR-505-3p, reportedly negatively regulates the expression of chemokine receptors CCR3, CCR4, and CXCR1 (Escate et al., 2018), suggesting that decreased miR-505-3p levels could enhance immune cell migration and tissue invasion. Thus, the miRNAs dysregulated by HIV infection in patients with active TB could contribute to excessive inflammation in HIV-related TB disease.

Dysregulation of miRNAs is a possible mechanism underlying HIV infection pathogenicity (Chiang et al., 2012). Furthermore, studies on peripheral blood mononuclear cells have demonstrated HIV-associated downregulation of miRNA expression (Houzet et al., 2008). Interestingly, miR-27b-3p, which is downregulated in HIV+/TB+ individuals, has been shown to suppress HIV replication (Chiang et al., 2012), and its levels are lower in individuals with viremic progression than in elite controllers (Egaña-Gorroño et al., 2014). The mechanisms by which HIV alters miRNA expression have not been fully elucidated, but it has been suggested that the viral regulatory protein Tat inhibits the capacity of the enzyme Dicer to process miRNA precursors (Sánchez-del Cojo et al., 2011).

Interestingly, our results also showed a pronounced effect on the snoRNA expression in individuals with active TB. The biological consequences of this finding are difficult to interpret, as there is limited data on the role of snoRNAs in the immune response to pathogens, including Mtb and HIV. A previous study by de Araujo et al. (2019) found one upregulated and one downregulated snoRNA in whole blood samples of patients with TB. In contrast, we observed differential expression of a range of snoRNAs in patients with active TB. The reasons for these discordant findings may depend on the methods used for identifying differentially expressed snoRNAs, i.e., sequencing vs. microarray, or the differences in method of filtering sncRNAs before analysis. Of note, snoRNA U104, whose expression level was decreased in subjects with TB in their study, was also significantly decreased in our HIV+/TB+ subjects. Furthermore, they found that snoRNA expression was downregulated in Mtb-infected peripheral blood mononuclear cells and M2 macrophages, but not in classically activated M1 macrophages. Classically activated M1 macrophages have a better capacity to limit mycobacterial growth than alternatively activated M2 macrophages (Rao Muvva et al., 2019). Whether downregulating snoRNA expression is a host defense mechanism driving the immune response toward effective clearance of infection, or whether it is induced by mycobacteria to evade host response is not clear. Interestingly, lower expression of snoRNAs U32A, U33, U34, and U35 in HIV-/TB- controls, has been linked to the diminished ability of fibroblasts to generate reactive oxygen species (Michel et al., 2011), raising the possibility that this could be a mechanism by which Mtb evades an immune mechanism. The role and mechanisms underlying the dysregulated expression of snoRNAs in TB and HIV infection warrant further studies.

Of note, we observed significant changes in sncRNA expression during treatment in TB and HIV coinfected individuals. This further supports the conclusion that the observed changes are triggered by these respective infections rather than by an unidentified confounder. Whether the observed changes in miRNA levels are related to ART or ATT cannot fully be clarified by the current study. However, since alterations in miRNA expression at baseline mainly were associated with the HIV infection, we consider it most likely that the longitudinal changes in miRNA are due HIV suppression through ART. The only significant correlation to CD4^+^ cell count was observed to be with the snoRNA U46 expression in coinfected individuals. Importantly, this correlation was not observed during the treatment, suggesting that this correlation is associated with HIV-related mechanisms other than the depletion of absolute CD4^+^ cell count. Since we could not reliably distinguish whether a correlation between a cell type and a sncRNA was due to actual expression of the sncRNA in that particular cell type, or by a separate effect of the HIV and/or TB infection status on cell counts and sncRNA levels, we opted against adjusting for peripheral blood cell counts in the statistical analysis. It is likely, however, that some of the sncRNA alterations noted in whole blood are related to different cell type compositions, rather than intracellular changes of sncRNA expression. Thus, further studies on the relation between sncRNA expression and specific cell populations are needed.

This study has certain limitations including the relatively small number of patients enrolled in the study. Furthermore, it should be noted that several of the differentially expressed miRNAs in the microarray were of low annotation confidence, and that microarray studies likely contain some false positive findings. However, the strong agreement between the microarray and qPCR results strengthens the conclusions inferred from the array data. The fact that the methods of TB diagnosis were different in the HIV+/TB+ and HIV-/TB+ groups is also a possible confounder, as the larger proportion of smear-positive subjects in the HIV-/TB+ group implies greater bacterial burden in the airway secretions. Still, a larger proportion of sputum smear positivity among HIV-negative individuals is expected due to the lower sensitivity of sputum smear microscopy in PWH (Esmail et al., 2018).

In summary, we observed distinct alterations in miRNA expression patterns associated with HIV coinfection among adults with active TB. These phenomena are likely to reflect dysregulated immune responses contributing to the pathogenesis of TB in PWH. In addition, the global downregulation of snoRNA in TB regardless of HIV status implies a role of these mediators in active TB. Further studies to elucidate the regulatory roles of miRNAs and snoRNAs in HIV-TB coinfection are required to understand how these mediators are involved in pathogenesis and to identify novel intervention targets.

## Data Availability Statement

The data presented in the study are deposited in the BioStudies repository, accession number S-BSST715 (https://www.ebi.ac.uk/biostudies/studies/S-BSST715).

## Ethics Statement

The study was approved by the National Research Ethics Review Committee, Addis Abeba, the Armauer Hansen Research Institute (AHRI/ALERT) Ethics Review Committee, Addis Abeba, Ethiopia, and the Regional Ethical Review Board at Lund University. Written informed consent was obtained from all the study participants.

## Author Contributions

OO: laboratory work, data analysis, and writing manuscript. FT: conceptualization, laboratory work, and revising and approving manuscript. RS and CR: planning of laboratory work, data analysis, revising, and approving manuscript. JM: conceptualization, planning of project, revising, and approving manuscript. MA, AA, and HY: laboratory work, revising, and approving manuscript. SS and PB: conceptualization, data analysis, revising, and approving manuscript. TB: data analysis, revising, and approving manuscript. MJ: conceptualization, data analysis, supervision of laboratory work, revising, and approving manuscript. All authors contributed to the article and approved the submitted version.

## Conflict of Interest

The authors declare that the research was conducted in the absence of any commercial or financial relationships that could be construed as a potential conflict of interest.

## Publisher’s Note

All claims expressed in this article are solely those of the authors and do not necessarily represent those of their affiliated organizations, or those of the publisher, the editors and the reviewers. Any product that may be evaluated in this article, or claim that may be made by its manufacturer, is not guaranteed or endorsed by the publisher.

## Footnotes

1
https://www.unaids.org/sites/default/files/media_asset/tuberculosis-and-hiv-progress-towards-the-2020-target_en.pdf

2
https://www.who.int/hiv/pub/guidelines/ethiopia_art.pdf

3
https://mirbase.org/
