# Supplementary material for: Expression of MicroRNAs Is Dysregulated by HIV While Mycobacterium tuberculosis Drives Alterations of Small Nucleolar RNAs in HIV Positive Adults With Active Tuberculosis
Source: Front Microbiol. 2022 Feb 22;12:808250. doi: 10.3389/fmicb.2021.808250 (PMC8920554; doi:10.3389/fmicb.2021.808250)
Supplement: Supplementary file 1 [file Table_1.docx]

| **Supplementary Table 1. Design of primers used for RT-qPCR** | | |
| --- | --- | --- |
|  | Forward | Reverse |
| miR-27b-3p | gcagttcacagtggctaag | tccagtttttttttttttttgcaga |
| miR-139-5p | gtctacagtgcacgtgtc | ccagtttttttttttttttactggag |
| miR-199a-5p | gcccagtgttcagactac | gtccagtttttttttttttttgaacag |
| snoRNA U46 | agtagggtgatgaaaaagaatcct | caagacggccacaaccac |
