# Supplementary material for: Expression of MicroRNAs Is Dysregulated by HIV While Mycobacterium tuberculosis Drives Alterations of Small Nucleolar RNAs in HIV Positive Adults With Active Tuberculosis
Source: Front Microbiol. 2022 Feb 22;12:808250. doi: 10.3389/fmicb.2021.808250 (PMC8920554; doi:10.3389/fmicb.2021.808250)
Supplement: Supplementary file 3 [file Image_1.pdf]

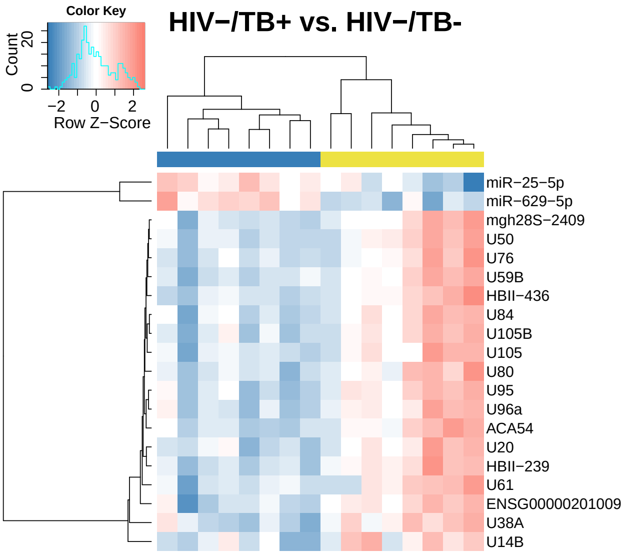

Supplementary figure 1. Hierarchical clustering analyses including HIV-/TB+ subjects and HIV-/TB- subjects. The 20 sncRNA that generated the smallest P values in Limma analysis from respective comparison. Microarray data median centred before analysis. Pearson's correlation used as distance matrix. Heatmap red colour indicate upregulated and blue colour downregulated sncRNA. Colour codes at the top represent infection status, HIV+/TB+ (red), HIV-/TB+ (blue) and HIV-/TB-(yellow). Probe HBII 438A also assigned to HBII-438B, probe U84 also assigned to ENSG00000263442/
