# Supplementary material for: Expression of MicroRNAs Is Dysregulated by HIV While Mycobacterium tuberculosis Drives Alterations of Small Nucleolar RNAs in HIV Positive Adults With Active Tuberculosis
Source: Front Microbiol. 2022 Feb 22;12:808250. doi: 10.3389/fmicb.2021.808250 (PMC8920554; doi:10.3389/fmicb.2021.808250)
Supplement: Supplementary file 4 [file Image_2.pdf]

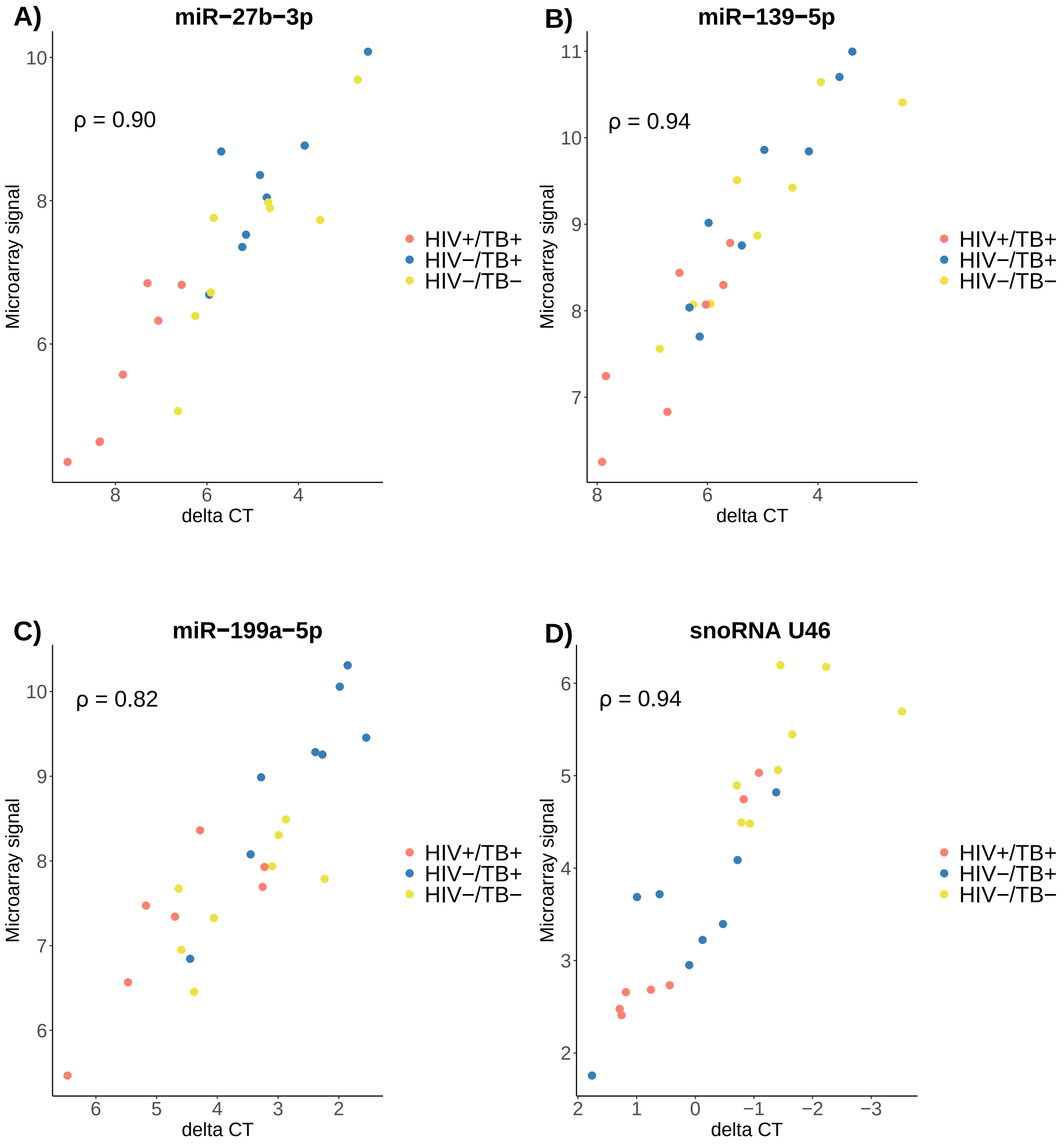

Supplementary Figure 2: Correlations between microarray signals and qPCR delta CT values for A) miR-27b-3p, B) miR-139-5p, C) miR-199a-5p and D) snoRNA U46. Colour code represent infection status, HIV+/TB+ (red), HIV-/TB+ (blue) and HIV-/TB- (yellow). Correlations calculated according to Spearman Rank correlation test.
