# Supplementary material for: Expression of MicroRNAs Is Dysregulated by HIV While Mycobacterium tuberculosis Drives Alterations of Small Nucleolar RNAs in HIV Positive Adults With Active Tuberculosis
Source: Front Microbiol. 2022 Feb 22;12:808250. doi: 10.3389/fmicb.2021.808250 (PMC8920554; doi:10.3389/fmicb.2021.808250)
Supplement: Supplementary file 5 [file Image_3.pdf]

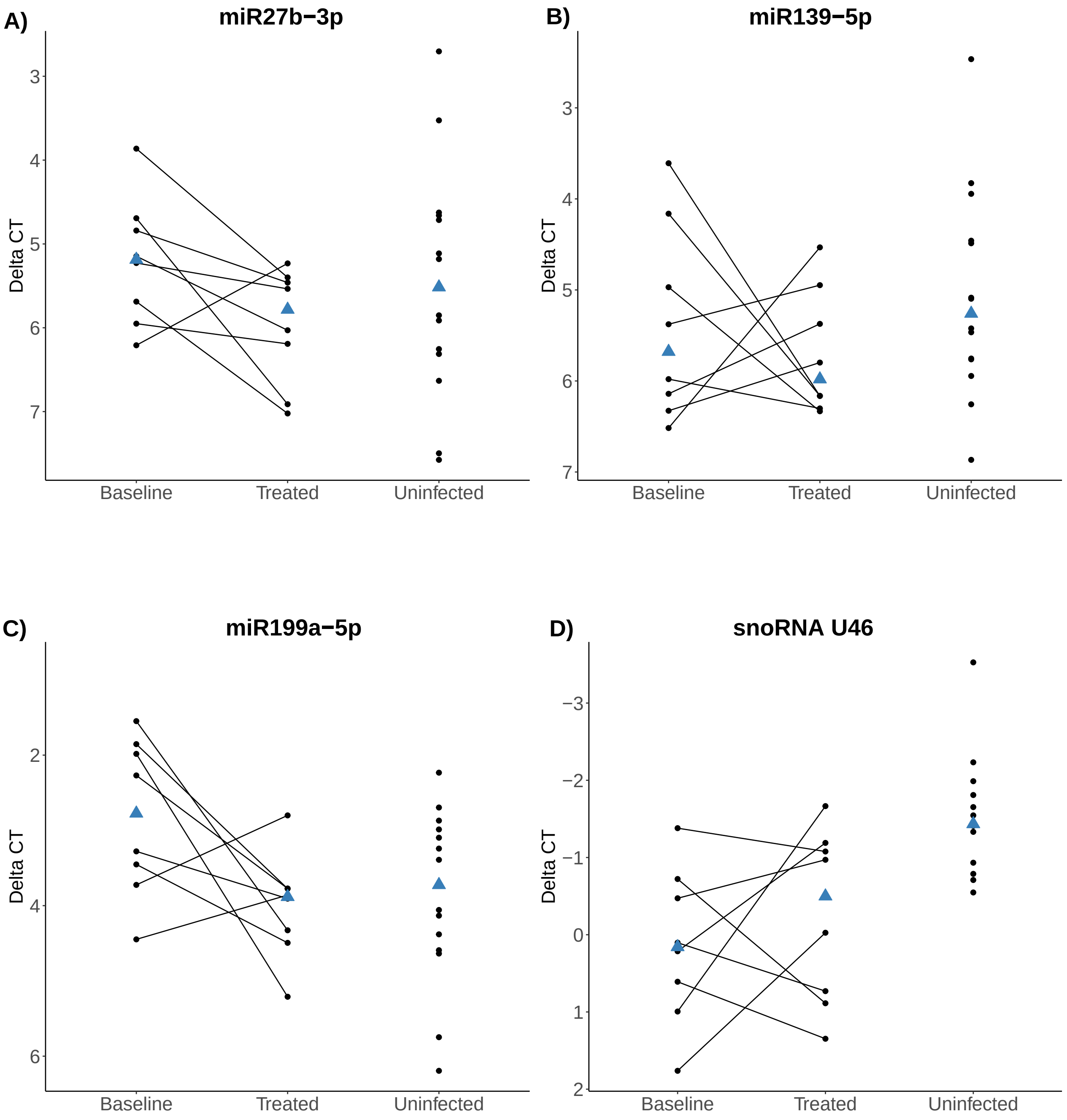

Supplementary Figure 3. Expression levels of A) miR-27 B) miR-139-5p C) miR-199a-5p D) snoRNA U46 in HIV-/TB+ subjects. Paired samples longitudinally followed before and during treatment connected with a line. HIV-/TB- control group included in all plots for comparison. Blue triangle indicating median value. No changes were statistically significant.
